# Diabetes prevalence and outcomes in hospitalized cardiorenal-syndrome patients with and without hyponatremia

**DOI:** 10.1186/s12882-020-02032-z

**Published:** 2020-09-10

**Authors:** Rainer U. Pliquett, Katrin Schlump, Andreas Wienke, Babett Bartling, Michel Noutsias, Alexander Tamm, Matthias Girndt

**Affiliations:** 1 Department of Internal Medicine II, Martin - Luther University Halle-Wittenberg, Halle (Saale), Germany; 2 Department of Nephrology & Diabetology, Carl-Thiem Hospital, Cottbus, Thiemstrasse 111, 03048 Cottbus, Germany; 3 Institute of Medical Epidemiology, Biometry and Informatics, Martin-Luther University Halle-Wittenberg, Halle (Saale), Germany; 4 University Clinic and Outpatient Clinic for Cardiac Surgery, Martin - Luther University Halle-Wittenberg, Halle (Saale), Germany; 5 University Clinic and Outpatient Clinic for Internal Medicine III, Martin-Luther University Halle-Wittenberg, Halle (Saale), Germany; 6 Department of Cardiology, University Mainz, Mainz, Germany

**Keywords:** Cardiorenal syndrome, Diabetes mellitus, Hyponatremia, Hypovolemia

## Abstract

**Background:**

Hyponatremia is known to be associated with a worse patient outcome in heart failure. In cardiorenal syndrome (CRS), the prognostic role of concomitant hyponatremia is unclear. We sought to evaluate potential risk factors for hyponatremia in patients with CRS presenting with or without hyponatremia on hospital admission.

**Methods:**

In a retrospective study, we investigated 262 CRS patients without sepsis admitted to the University Hospital Halle over a course of 4 years. CRS diagnosis was derived from an electronic search of concomitant diagnoses of acute or chronic (NYHA 3–4) heart failure and acute kidney injury (AKIN 1–3) or chronic kidney disease (KDIGO G3-G5_nonD_). A verification of CRS diagnosis was done based on patient records. Depending on the presence (Na < 135 mmol/L) or absence (Na ≥ 135 mmol/L) of hyponatremia on admission, the CRS patients were analyzed for comorbidities such as diabetes, presence of hypovolemia on admission, need for renal replacement therapy and prognostic factors such as in-hospital and one-year mortality.

**Results:**

Two hundred sixty-two CRS patients were included in this study, thereof, 90 CRS patients (34.4%) with hyponatremia (Na < 135 mmol/L). The diabetes prevalence among CRS patients was high (> 65%) and not related to the serum sodium concentration on admission. In comparison to non-hyponatremic CRS patients, the hyponatremic patients had a lower serum osmolality, hypovolemia was more prevalent (41.1% versus 16.3%, *p* < 0.001). As possible causes of hypovolemia, diarrhea, a higher number of diuretic drug classes and higher diuretic dosages were found. Hyponatremic and non-hyponatremic CRS patients had a comparable need for renal-replacement therapy (36.7% versus 31.4%) during the hospital stay. However, after discharge, relatively more hyponatremic CRS patients on renal replacement therapy switched to a non-dialysis therapy regimen (50.0% versus 22.2%). Hyponatremic CRS patients showed a trend for a higher in-hospital mortality (15.6% versus 7.6%, *p* = 0.054), but no difference in the one-year mortality (43.3% versus 40.1%, *p* = 0.692).

**Conclusions:**

All CRS patients showed a high prevalence of diabetes mellitus and a high one-year mortality. In comparison to non-hyponatremic CRS patients, hyponatremic ones were more likely to have hypovolemia, and had a higher likelihood for temporary renal replacement therapy.

## Background

Cardiorenal syndrome (CRS), defined as concurrent acute or chronic heart failure (CHF) and acute kidney injury (AKI) or chronic kidney disease (CKD), is associated with a poor prognosis as the presence of renal disease is a stronger predictor for death CHF patients than the degree of reduced left ventricular ejection fraction (LVEF) [1–3]. In CHF patients, an AKI is a leading cause for an acute decompensation [4]. The term acute “renocardiac” syndrome [5] acknowledges this pathophysiology. A more practical definition [6] differentiates between a severe and a stable, chronic CRS, regardless of the pathophysiology. Animal models of CRS demonstrated the bidirectional nature, where the failure of the one organ system adversely affects the function of the other [7]. The consideration of hypo- and hypervolemic states may further differentiate CRS on clinical grounds [8].

Arginine-vasopressin (AVP) is released from the posterior pituitary gland upon changes in osmolality and neurally via baroreceptor-mediated activation due to intraarterial hypovolemia or arterial hypotension, thus representing an adaptive mechanism in CHF [9]. AVP acts on vascular smooth muscle cells via vasopressin 1A receptors and on renal collecting-duct cells via vasopressin 2 receptors. In CHF, AVP release occurs in a linear fashion to the functional New York Heart Association (NYHA) class [10]. On the flip side, an inappropriate release of AVP may lead to a dilutional hyponatremia and to a deleterious outcome in CHF [11]. Conversely, hypovolemia, e.g. due to diuretic medication, may lead to hypotension as a trigger for an appropriate non-osmotic AVP release, possibly leading to a dilutional hyponatremia as well. Except for sodium-glucose transporter-2 inhibitors in type-2 diabetes patients [12] and in patients with heart failure with reduced LVEF [13], the use of diuretics has not reduced mortality in CHF. Counter-regulators of hypovolemia such as an appropriate AVP release and a sympathetic-nervous-system activation may account for this lack of benefit.

In the present study, possible contributing factors for hyponatremia such as presence of hypovolemia, use of diuretics, sodium deficiency and hyperglycemia [14] were obtained from patient records. Furthermore, the prevalence of hypoglycemia on admission in the diabetics among CRS patients was also analyzed. This is because hypoglycemia has been found to be linked to excess mortality in cardiovascular outcome studies [15], and a poorly-controlled diabetes mellitus is usually characterized by post-hypoglycemic hyperglycemic episodes, which, in turn, may depress serum sodium [14]. As a correlate to the fact that hyponatremic CHF patients have a poorer outcome [16], we hypothesized that hyponatremia in CRS worsens patient outcome.

## Methods

### Patients and study design

In a retrospective observational study, all CRS patients admitted to the University Hospital Halle (Department of Internal Medicine II) over a 4-year period were included according to in- and exclusion criteria. The institutional review board of the Martin-Luther-University Halle-Wittenberg approved this study (file number 2013–92).

#### Inclusion criteria

Acutely decompensated or CHF (NYHA class 3 or 4) accompanied by an AKI, stage 1–3 (AKI Network (AKIN) classification [17]) or by CKD stage 3 - 5_nonD_ according to Kidney Disease: Improving Global Outcomes (KDIGO) classification [18].

#### Exclusion criteria

Diagnosis of a malignant tumor within 5 years except non-melanoma skin tumor, the need for peritoneal or hemodialysis prior to admission or index hospitalization, sepsis with procalcitonin plasma levels > 10 ng/mL.

### Diagnosis of cardiorenal syndrome

CRS diagnosis derived from a search in the electronic medical records of the hospital patient-management software using codes for acute or CHF and AKI or CKD according to the *International Statistical Classification of Diseases and Related Health Problems* (ICD 10th edition) [19]. In addition, a manual verification of CRS diagnosis based on patients’ records was mandatory. The first hospitalization in the study period, where heart failure and kidney disease were coded concomitantly, was regarded as index hospitalization. To differentiate between CKD and AKI, the available serum creatinine up to 3 months prior to the index hospitalization and serum creatinine at discharge were taken into account. AKI classification [16] relied on changes in serum creatinine. Changes in urinary output were not considered. Estimated glomerular filtration rate (eGFR) [20] was provided, when creatinine prior to index hospitalization and/or creatinine at discharge showed a variance of less than 26.5 μmol/l, as a steady state of serum creatinine is a prerequisite for eGFR calculation. Echocardiography recordings of the index hospitalization were reevaluated by a cardiologist to ascertain the diagnosis of heart failure and/or to differentiate the diagnosis heart failure with reduced (HFrEF) or with preserved ejection fraction. The biplane calculation of LVEF was mandated to qualify for either form of CHF and the diastolic ventricular function was assessed using the E/E´ ratio.

### Analysis

Analysis relied on patient cohorts with presence (Na < 135 mmol/L) or absence of hyponatremia (Na ≥ 135 mmol/L) on admission. For further analysis, the hyponatremia cohort was divided into subgroups of mild (Na < 135 mmol/L, Na ≥ 130 mmol/L), moderate (Na < 130 mmol/L, Na ≥ 125 mmol/L), and severe hyponatremia (Na < 125 mmol/L). Information on the presence or absence of hypovolemia at admission (recorded findings of “exsiccosis”, “dehydratation” or “hypovolemia” based on clinical signs including reduced tongue moisture and/or reduced skin turgor and/or sonographic evidence for an inferior vena cava collapsibility [21]), the use and, if applicable, the dosage of diuretics, presence or absence of diarrhea on admission and of diabetes mellitus were collected from patient records. In case of diabetes, the presence or absence of a hypoglycemic episode on admission was determined. Hypoglycemia was assumed, if capillary blood glucose was < 3.9 mmol/L. Additional laboratory results gathered within 24 h hours after admission were serum procalcitonin, C-reactive protein, creatinine, urea, brain natriuretic peptide, hemoglobin A1c (HbA1c), osmolality, fasting capillary-blood glucose, urinary sodium concentration and urinary sodium excretion over 24 h. If laboratory values were below the lower detection limit, then the lower detection limit was used for analysis.

Outcome parameters were length of the hospital stay, in-hospital and one-year mortality, need for temporary (< 1 year) and chronic (> 1 year) peritoneal or hemodialysis. Resident’s registration offices of patients’ home towns were contacted to provide information on the date of death, if applicable, for up to 2 years after discharge in index hospitalization.

### Statistics

Data were given as mean with standard deviation or as the absolute number and percentage of all patients. The Kolmogorov-Smirnov test was used to check for normal distribution of the values. Differences between the mean values of two evaluation groups have been checked by Student’s t test (parametric data) or Mann-Whitney test (non-parametric data) and of more than two evaluation groups by the ANOVA test (parametric data) or Kruskal-Wallis test (non-parametric data) with post-hoc tests (Tukey or Dunn, where appropriate). Cox regression analysis was used for the determination of in-hospital and one-year mortality, Kaplan-Meier method with log-rank test for survival analysis. All data calculations were performed by use of the SPSS software (version 21; IBM Corp., Armonk, New York, USA). Graphs were displayed using the Graphpad software (Prism 8, La Jolla, California, USA).

## Results

Three hundred eighty-six data sets of CRS patients were identified when analyzing electronic medical records using the prespecified diagnosis codes for the study period. After a manual verification, 124 patients were excluded for one or more applicable exclusion criteria: sepsis (*n* = 7), malignancies (*n* = 47), an ongoing or prior hemodialysis treatment (*n* = 63), or absence of CHF (*n* = 11). The characteristics of the included 262 CRS patients with and without hyponatremia on admission are provided in Table 1. Of note, the hyponatremia cohort had a higher proportion of female patients.

**Table 1:** Characteristics of 262 CRS patients with and without hyponatremia at hospital admission

	CRS patients with hyponatremia^**a**^				CRS patients without hyponatremia^**a**^				
	% n	n	mean ± SD	n^b^	% n	n	mean ± SD	n^b^	*p* value
**Patient characteristics**									
Men (n)	36.7	33		90	52.3	90		172	0.019
Women (n)	63.3	57		90	47.7	82		172	
Age (years)		90	74.0 ± 13.0	90			73.8 ± 13.0	172	0.768
Body mass index (kg/m^2^)		41	30.2 ± 8.1	90		98	30.3 ± 7.3	172	0.709
Hypovolemia	41.1	37		90	16.3	28		172	< 0.0001
Diarrhea (n)	17.8	16		90	5.2	9		172	0.002
Classes of oral diuretics per patient (n)		88	1.6 ± 1.0	90		161	1.3 ± 0.7	172	0.003
Torasemide (mg/d)		68	23.1 ± 49.0	90		57	19.2 ± 28.0	172	0.612
Furosemide (mg/d)		70	34.4 ± 135.8	90		62	1.9 ± 7.9	172	0.003
Hydrochlorothiazide (mg/d)		71	7.7 ± 11.5	90		57	2.2 ± 6.3	172	0.002
Xipamide (mg/d)		67	4.3 ± 16.4	90		62	0.8 ± 3.3	172	0.159
Spironolactone (mg/d)		69	13.8 ± 38.2	90		63	5.0 ± 17.9	172	0.109
Eplerenone (mg/d)		70	1.1 ± 5.1	90		63	1.4 ± 5.6	172	0.707
**Laboratory parameters**									
Na (serum, mmol/L)		90	129.6 ± 5.1	90		172	139.4 ± 3.0	172	< 0.0001
Na at discharge (serum, mmol/L)		23	136.5 ± 4.3	75		16	139.0 ± 3.9	136	< 0.0001
Na (urine, mmol/L)		37	68.2 ± 34.5	90		76	78.3 ± 30.0	172	0.113
Na (collecting urine, mmol/24 h)		31	191.0 ± 163.8	90		63	185.5 ± 109.2	172	0.477
Urea (serum, mmol/L)		87	26.3 ± 14.6	90		160	22.4 ± 16.1	172	0.006
Cystatin C (serum, mg/mL)		25	2.7 ± 1.0	90		46	2.7 ± 1.1	172	0.827
Creatinine prior to hospitalization (serum, μmol/L)		53	148.8 ± 61.3	90		96	171.1 ± 107.0	172	0.251
Creatinine (serum, μmol/L)		90	300.7 ± 205.8	90		172	285.5 ± 256.1	172	0.147
Osmolality, calculated (serum, mosm/kg)		52	295.6 ± 22.2	90		93	308.4 ± 18.9	172	0.0002
C-reactive protein (serum, mg/L)		89	57.8 ± 75.0	90		172	54.5 ± 80.4	172	0.308
Procalcitonine (serum, pg/mL)		24	1.7 ± 2.6	90		38	1.0 ± 1.9	172	0.680
**Renal and cardiac parameters**									
Acute kidney injury	71.1	64		90	45.9	79		172	
AKIN 1	48.4	31		64	31.6	25		79	
AKIN 2	11.0	7		64	15.2	12		79	
AKIN 3	40.6	26		64	53.2	42		79	
Chronic kidney disease	28.9	26		90	54.1	93		172	
KDIGO 1–3	42.3	11		26	52.6	49		93	
KDIGO 4	19.2	5		26	23.7	22		93	
KDIGO 5	38.5	10		26	23.7	22		93	
Estimated glomerular filtration rate, if steady state (mL/min/1.73 m^2^)		26	27.9 ± 19.8	90		93	31.7 ± 20.1	172	0.403
arteriovenous fistula, preexisting (n)	5.5	5		90	14.5	25		172	0.017
Left ventricular ejection fraction (%)		56	48.5 ± 12.7	90		102	44.4 ± 13.4	172	0.046
Brain natriuretic peptide (serum, pg/mL)		56	1321.0 ± 1851.0	90		117	1319.0 ± 1725.0	172	0.940
**Diabetes-related parameters**									
Diabetes mellitus (n)	66.7	60		90	65.7	113		172	0.892
- insulin- dependent (n)	65.0	39		60	66.4	75		113	0.973
- oral antidiabetics (n)	8.3	5		60	11.5	13		113	0.608
- diet alone (n)	26.7	16		60	22.1	25		113	0.574
Glucose on admission, non-fasting (capillary blood, mmol/L)		51	9.6 ± 6.6	90		96	7.7 ± 3.5	172	0.063
Glucose, fasting (capillary blood, mmol/L)		15	7.5 ± 2.6	90		27	6.7 ± 1.7	172	0.209
HbA1c (%)		28	7.6 ± 2.6	90		47	6.6 ± 0.8	172	0.235
Hypoglycemia (symptomatic) on admission (n)	8.9	8		90	4.6	8		172	0.184
**Outcome parameters**									
Hospitalization (days)			16.5 ± 11.0	76		159	18.5 ± 16.5	172	0.905
new-onset hemodialysis (in hospital, n)	36.7	33		90	31.4	54		172	0.409
chronic dialysis^c^ (n)	19.6	10		51	27.0	27		103	0.104
Death in hospital (n)	15.6	14		90	7.6	13		172	0.054
One-year mortality (n)	43.3	39		90	40.1	69		172	0.692

^a^ hyponatremia at a serum Na concentration of < 135 mmol/L

^b^ final number of CRS patients subjected to statistical analysis. This number can be lower than the maximum number due to the lack of data

^c^ hemodialysis or peritoneal dialysis

### Cardiac and renal function in CRS patients

**Fig. 1:**
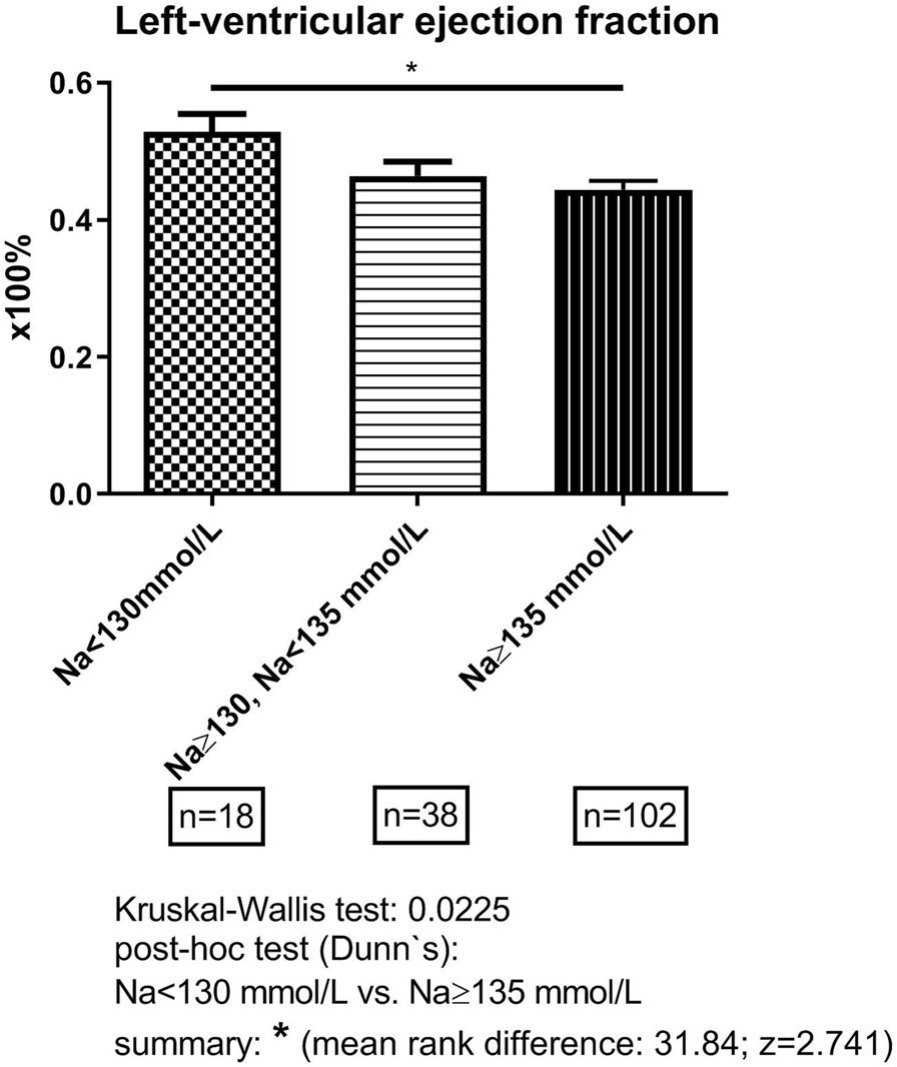
Group-wise data of left-ventricular ejection fraction (mean ± standard deviation) in cardiorenal-syndrome patients with presence or absence of mild (Na < 135 mmol/L, Na ≥ 130 mmol/L) or moderate-to-severe hyponatremia (Na < 130 mmol/L). Numbers indicate the number of patients with available information

LVEF was slightly higher in CRS patients with hyponatremia on admission than without (Table 1). CRS patients with moderate-to-severe hyponatremia on admission had a more preserved systolic function in comparison to the ones without hyponatremia or mild hyponatremia on admission (Fig. 1). Diastolic left ventricular dysfunction was highly prevalent in both cohorts (98.9% in hyponatremic, 98.3% in non-hyponatremic CRS patients). No echocardiography follow-up exams were available. An AKI occurred in more hyponatremic than non-hyponatremic CRS patients, while CKD was less frequently diagnosed in hyponatremic versus non-hyponatremic ones (Table 1). Prior to index hospitalization, an arterio-venous fistula as a preemptive vascular access was placed in a larger proportion of non-hyponatremic CRS patients than hyponatremic ones (Table 1).

### Prevalence and causes of hyponatremia in CRS patients

**Fig. 2:**
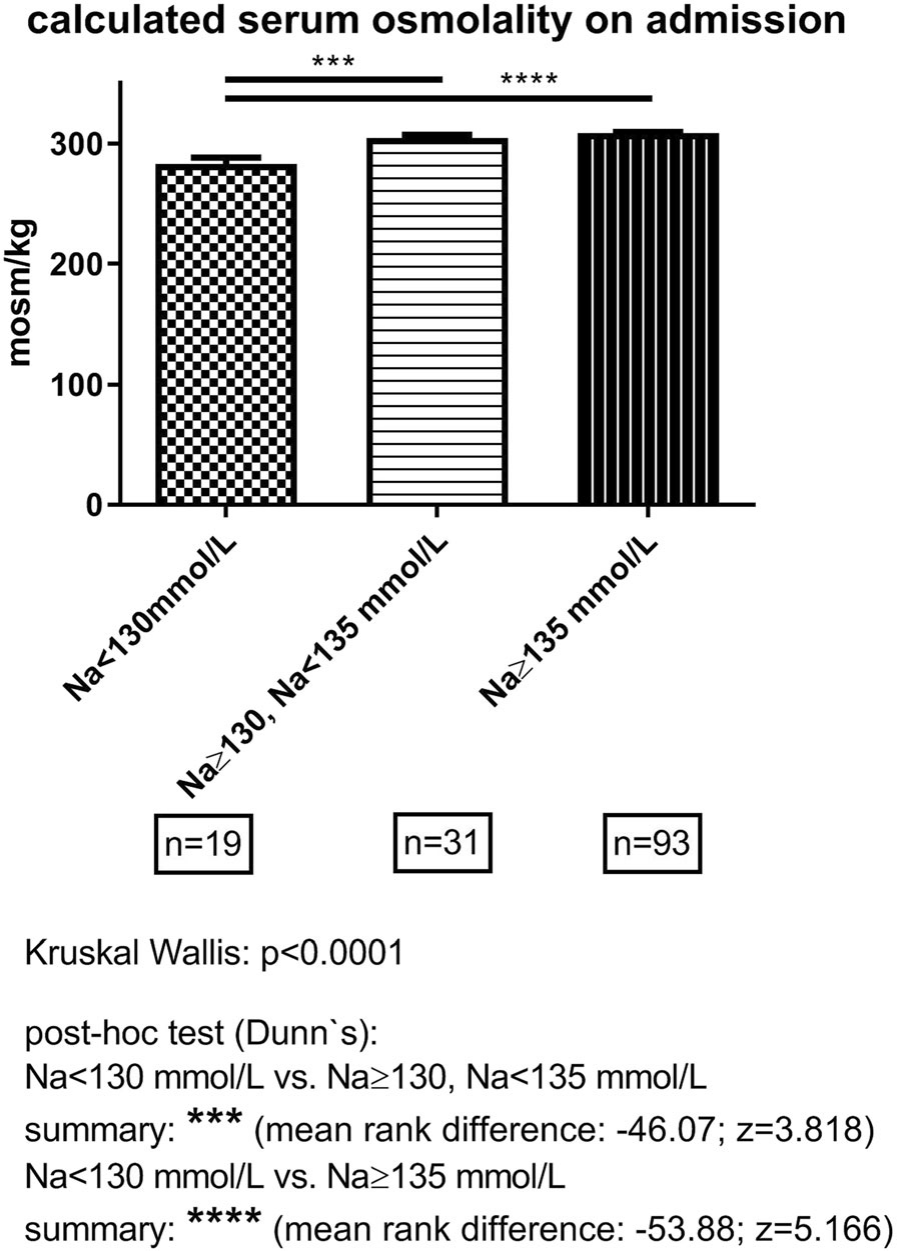
Group-wise display of calculated serum osmolality on admission according to presence or absence of mild (Na < 135 mmol/L, Na ≥ 130 mmol/L) or moderate-to-severe (Na < 130 mmol/L) hyponatremia. The following formula was used: serum osmolality = 2x Na (mmol/L) + Urea (mmol/L) + Glucose (mmol/L). Numbers indicate the available calculated results per group

On admission, 34.4% of CRS patients presented with hyponatremia. Among them, 9 (3.4%) patients had a severe, 22 (8.4%) patients a moderate, and 59 (22.5%) patients a mild hyponatremia on admission. In 7 (2.7%) non-hyponatremic CRS patients, a hypernatremia (maximum Na: 150 mmol/L) was found. As Fig. 2 demonstrates, hyponatremia correlated with serum osmolality in terms of a hyposmolar hyponatremia. A reduced urinary-sodium concentration (< 30 mmol/L) was found in 5 (5.6%) patients of the hyponatremic cohort versus 6 (3.5%) patients of the non-hyponatremic cohort. Likewise, collecting urine measurements showed a reduced urinary sodium excretion of less than 100 mmol/d (equaling 6 g/d sodium chloride) in 13 or 14.4% of hyponatremic versus 14 or 8.1% of non-hyponatremic CRS patients. As a contrasting finding, urinary sodium wasting (sodium excretion of > 300 mmol/d or > 18 g/d sodium chloride) was seen more often in the hyponatremia (26%) than in the non-hyponatremia cohort (13%).

**Fig. 3:**
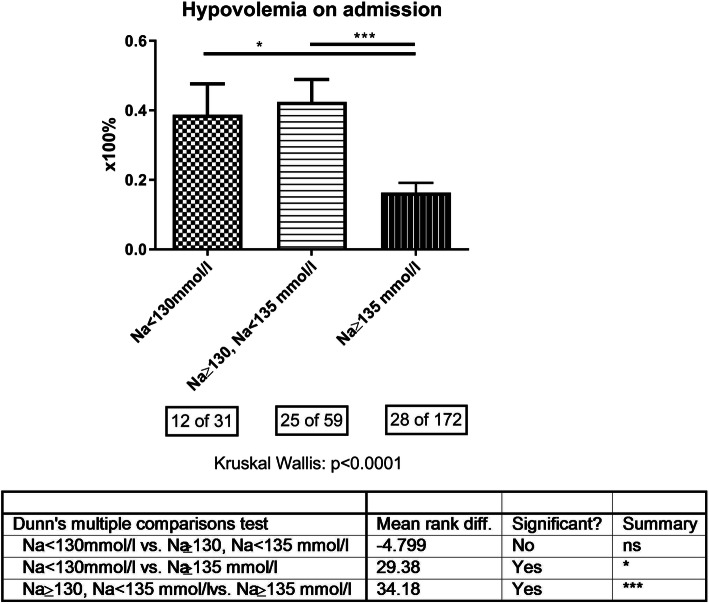
Group-wise display of clinical signs of hypovolemia on admission according to presence or absence of mild (Na < 135 mmol/L, Na ≥ 130 mmol/L) or moderate-to-severe (Na < 130 mmol/L) hyponatremia. Numbers indicate the absolute number of patients with hypovolemia and the total number of patients with available information

Hypovolemia was more frequently encountered in hyponatremic than in non-hyponatremic CRS patients (Fig. 3). Thee prevalence of diarrhea, the number of prescribed diuretic drug classes and, if applicable, the dosages of hydrochlorothiazide and furosemide were the possible underlying reasons accounting for the observations made. (Table 1). Diarrhea on admission was more frequently present in CRS patients both with mild (9 of 59 or 15.3%) and moderate-to-severe hyponatremia (7 of 31 or 22.6%) than in non-hyponatremic ones (9 of 172 or 5.2%, *p* = 0.0025).

### High prevalence of type-2 diabetes among CRS patients

Prevalence of diabetes mellitus on admission was equally high in CRS patients with (66.7%) versus CRS patients without hyponatremia (65.7%) on admission, while the prevalence in the background population of the hospital referral region was 12% [22]. The majority of diabetic CRS patients had a type-2 diabetes, except for 10 (14.4%) patients of the hyponatremia cohort and 13 (11.5%) of the non-hyponatremia cohort who had a type-1 diabetes, a steroid-related or a new-onset diabetes after transplant. A steroid therapy, either via oral or inhalation route, was applied in 24.7% of hyponatremic and in 28.8% of non-hyponatremic CRS patients respectively. An immunosuppressive regimen for a functioning kidney transplant was applied in 4.4% versus 3.5% of hyponatremic versus non-hyponatremic CRS patients. The proportion of insulin-dependent diabetes, the percentage of patients presenting with a symptomatic hypoglycemia on admission (Table 1) and, if applicable, the cumulative daily insulin dose (49.7 ± 47.7 units/d versus 52.1 ± 37.4 units/d, *p* = 0.369), were not different between CRS patients with and without hyponatremia. Likewise, the average insulin dose per body weight (0.64 ± 0.66 units/kg versus 0.59 ± 0.33 units/kg) was not different between hyponatremic versus non-hyponatremic CRS patients. With regard to non-insulin therapy of type-2 diabetes, only one diabetes patient of the hyponatremia cohort was treated with incretin mimetics in addition to insulin, none was treated with sodium-glucose-transporter-2 inhibitors.

### Outcomes of CRS patients with and without hyponatremia

**Fig. 4:**
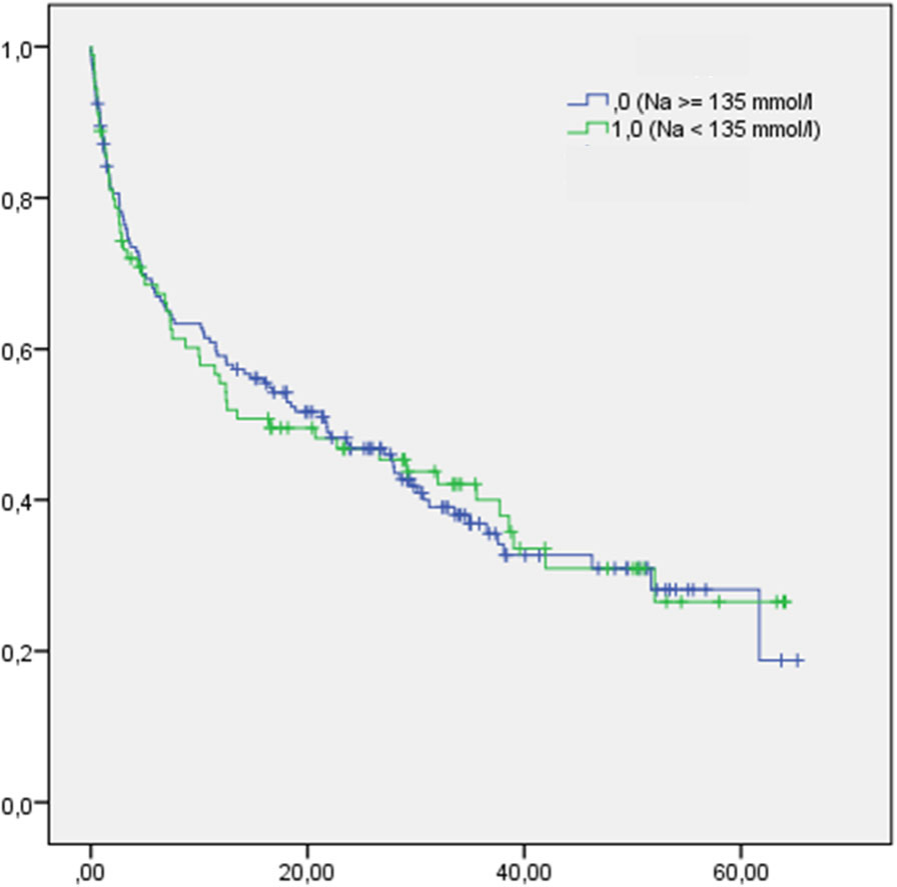
Survival data of CRS patients with (Na < 135 mmol/L) and without (Na ≥ 135 mmol/L) hyponatremia

The length of hospital stay (Table 1) and survival (Fig. 4) were not different between hyponatremic versus non-hyponatremic CRS patients. In non-hyponatremic CRS patients, median survival was 17.4 (range: 0.004 to 65.2) months, while it was, 12.5 (range: 0.049 to 63.9) months in mildly hyponatremic, and 13.5 (range: 0.278 to 64.1) months in moderate-to-severe hyponatremic CRS patients. One-year mortality was high both in CRS patients with (43.3%) and without (40.1%) hyponatremia on admission. Lastly, in-hospital mortality was higher among hyponatremic (15.6%) than among non-hyponatremic (7.6%) patients (Table 1).

Age on admission (hazard ratio: 1.05; *p* < 0.001).6 and male sex (hazard ratio: 1.48; *p* = 0.02) were predictors for mortality. Even though one-year mortality was not different between groups, a subgroup analysis with a cut off at an age of 60 years was performed. A comparable one-year mortality was found in CRS patients being older than 60 years (45.3% versus 45.4% in hypo- versus non-hyponatremic CRS patients). However, hyponatremic CRS patients being less than 60 years had a higher one-year mortality (40.6%) than its non-hyponatremic counterparts (9.6%). At discharge, 30.7% of the initial hyponatremia cohort and 11.8% of the initial non-hyponatremia cohort presented with mild or moderate hyponatremia. Within 1 year after discharge, 8 (10.7%) persistently hyponatremic patients of the initial hyponatremic cohort and 6 (4.4%) hyponatremic patients of the initial non-hyponatremia cohort died.

As for renal replacement therapy, hemodialysis therapy was chosen, if needed, during index hospitalization (Table 1). By discharge, the proportion of patients on hemodialysis was comparable between groups: 26 (34.2%) surviving patients of the hyponatremia cohort and 49 (30.8%) surviving patients of the non-hyponatremia cohort were placed on an intermittent hemodialysis schedule (thrice-a-week). None of the hyponatremia cohort, but 2 (1.2%) patients of the non-hyponatremia cohort switched to peritoneal dialysis within 1 year after discharge. Among CRS patients without renal-replacement therapy during index hospitalization, 1 out of 76 (or 1.3%) in the hyponatremia cohort and 3 out of 159 (or 1.9%) in the non-hyponatremia cohort initiated hemodialysis after discharge during the following year. Within 1 year after discharge, 50.0% of hyponatremic versus 22.2% of non-hyponatremic CRS patients on renal replacement therapy switched to a conservative therapy regimen without hemo- or peritoneal dialysis.

## Discussion

As main findings, CRS patients included in this study had both a high prevalence of diabetes mellitus and a high one-year mortality, regardless of serum sodium concentration on admission. So far, this is the first report showing a diabetes prevalence of more than 65% in consecutively hospitalized CRS patients. However, there was no relation between hyperglycemia and hyponatremia in CRS patients. The finding of an exceedingly increased one-year mortality of CRS patients is in line with previous evidence [23]. Therefore, as hospitalizations for CRS greatly increased by up to 17% in internal-medicine departments in recent years [24], it is of paramount importance to improve the overall outcome of patients with CRS.

### Diabetes mellitus as a double-organ risk

Regarding diabetes mellitus in CRS, diabetes-related vascular sequelae may translate into cardiac and renal dysfunction in terms of a CRS, type 5 [5]. In the literature, CHF patients with diabetes mellitus were shown to have a higher CKD prevalence than CHF patients without diabetes [25]. Likewise, in a recent retrospective study, more than 50% of CRS patients with incident peritoneal-dialysis therapy had diabetes mellitus [26]. The current study showed that the mean cumulative daily insulin dose per body weight was higher than expected suggesting an insulin resistance or, alternatively, an over-dosage of insulin. Supporting the first scenario, roughly one quarter of CRS patients of both cohorts was on a steroid therapy. In addition, inflammatory parameters were elevated on admission. As for the latter scenario, the number of symptomatic hypoglycemic episodes on hospital admission was remarkably high in both cohorts. Given a pro-hypoglycemic role of renal dysfunction, an insulin-minimizing strategy is warranted in CRS. In addition, as for the proven nephro- and cardio-protection by both incretin mimetics [27] and sodium-glucose cotransporter-2 inhibitors [28] for type-2 diabetes patients, at least incretin mimetics could be used as an alternative to insulin in CRS patients with type-2 diabetes and without need for renal replacement therapy [27]. In acute CRS, sodium glucose cotransporter-2 inhibitors should be considered, when renal function has fully recovered. This drug class has shown a prognostic benefit even in HFrEF patients without diabetes mellitus [13].

### Hypovolemia as a main cause of hyponatremia in hospitalized CRS patients

Although hyponatremia was shown to be multifactorial, in more than one third of hyponatremic CRS patients, there were signs of hypovolemia on admission. Hypovolemia was substantiated by a more frequent history of diarrhea, by more prescribed diuretic drug classes and by higher dosages of prescribed diuretics in the hyponatremia cohort. It remains unclear, whether or not the difference in cardiac systolic function between hyponatremic and non-hyponatremic CRS patients (Fig. 1) was due to a lesser end-diastolic left-ventricular pressure during hypovolemia. The extensive use of diuretics has been established as a leading cause for hypovolemia in CRS patients of this study. Here, the dose combinations of several diuretics appear to bear an increased risk for hypovolemia-related hyponatremia or for hyponatremia as a side effect of diuretics. In addition, the use of a diuretic combination therapy favors a rise in serum urea [29]. Besides hypovolemia, the percentage of patients with excessive sodium excretion in 24-h collecting-urine measurements was twice as high in hyponatremic versus non-hyponatremic CRS patients. This result is in line with either renal sodium wasting or increased sodium-chloride consumption. The latter was excluded for the patients of the current study. Conversely, the use of loop diuretics as a cause for sodium depletion is highly likely, as the prescribed furosemide dosage was higher among hyponatremic CRS patients in comparison to non-hyponatremic ones. In addition to sodium depletion, loop diuretics induce a hypovolemia-triggered, appropriate AVP activation [30].

In case of hypervolemia-associated hyponatremia, free-water excess and/or diuretic resistance may apply as pathomechanisms for hyponatremia. Among hypervolemic CRS patients included in this study, a further differentiation based on chloremia was not performed. A diuretic resistance is possible in cases of hypochloremia due to less active chloride-sensitive kinases such as the serine/threonine kinase WNK1 [31]. However, on clinical grounds, a diuretic resistance can be assumed in all patients with new-onset hemodialysis. Comparable proportions of CRS patients with and without hyponatremia experienced new-onset hemodialysis though.

### Cardiorenal syndrome with hyponatremia as an acute, amenable condition

The likelihood for a temporary, not chronic renal-replacement therapy after discharge was higher in the hyponatremia versus non-hyponatremia cohort (50.0% versus 22.2%). These results are compatible with an acute and amenable condition like AKI or acutely decompensated heart failure in hyponatremic CRS patients. This view is supported by the overall higher incidence of AKI among hyponatremic compared to non-hyponatremic CRS patients, and by the fact that more non-hyponatremic than hyponatremic CRS patients had a preemptive arterio-venous fistula signifying a rather advanced CKD condition there. In retrospect, the placement of arterio-venous fistulae in CRS patients appears to be questionable, as the shunt volume may aggravate heart failure [32].

### Outlook and study constraints

In retrospective, observational studies, causative relationships cannot be established. However, from the current study, a prognostic role for hyponatremia in CRS patients is questioned. With regard to hyponatremia at discharge, the number of study participants was too small to assess a possible prognostic role. More pathophysiological studies are needed to further dissect the underlying mechanisms of hyponatremia in CRS. In addition, randomized clinical trials may help identify new treatment approaches and/or gauge the effect of existing ones for CRS with and without diabetes mellitus. An individualized ambulatory patient care needs to focus on euvolemia as a therapy goal. There, a careful use of diuretics and an adequate diabetes control, if applicable, are crucial. As both peritoneal dialysis [33, 34] and hemodialysis [35] may improve the functional capacity in CHF symptoms, it is subject to future studies to compare these therapy modalities in CRS patients with and without diabetes mellitus.

## Conclusions

As a main result, this study confirms the high death toll among CRS patients both with and without hyponatremia on admission. The finding of a very high prevalence of diabetes mellitus among all CRS patients may increase the awareness for CRS, when diagnosis of diabetes mellitus has been established. Lastly, hyponatremia in CRS patients of this study was shown to be multifactorial, and hyponatremic CRS patients more frequently had an acute decompensation of heart and/or kidney function.

## Data Availability

The datasets used and/or analyzed during the current study are available from the corresponding author on reasonable request.

## Abbreviations

AKIN: Acute kidney injury network
AVP: Arginine vasopressin
CHF: Chronic heart failure
CRS: Cardiorenal syndrome
d: Day(s)
eGFR: Estimated glomerular filtration rate
HbA1c: Hemoglobin A1c
HFrEF: Heart failure with reduced ejection fraction
KDIGO: Kidney Disease: Improving Global Outcomes
LVEF: Left ventricular ejection fraction
Na: Sodium
NYHA: New York Heart Association
